# Managing Free-Range Laying Hens—Part B: Early Range Users Have More Pathology Findings at the End of Lay but Have a Significantly Higher Chance of Survival—An Indicative Study

**DOI:** 10.3390/ani10101911

**Published:** 2020-10-18

**Authors:** Terence Zimazile Sibanda, Cormac J. O’Shea, Jessica de Souza Vilela, Manisha Kolakshyapati, Mitchell Welch, Derek Schneider, Jodi Courtice, Isabelle Ruhnke

**Affiliations:** 1 School of Environmental and Rural Science, Faculty of Science, Agriculture, Business and Law, University of New England, Armidale, NSW 2351, Australia; desouzavilelajessica@gmail.com (J.d.S.V.); mkolaks2@une.edu.au (M.K.); iruhnke@une.edu.au (I.R.); 2 School of Biosciences, University of Nottingham, Nottingham LE12 5RD, UK; Cormac.O'Shea@nottingham.ac.uk (C.J.O.); mwelch8@une.edu.au (M.W.); dschnei5@une.edu.au (D.S.); 3 Division of Research and Innovation, University of Southern Queensland, Toowoomba, QLD 4350, Australia; jodi.courtice@gmail.com

**Keywords:** behaviour, egg, feather cover, health, housing, mortality, non-caged, parasites, poultry, roundworms, tapeworms, welfare

## Abstract

**Simple Summary:**

Free-range facilities may present a biosecurity risk in some situations, but range use has also been associated with better hen welfare. We investigated the association between early-life range use (when hens were 18–21 weeks of age) and hen survival during the entire housing period as well as various health and welfare parameters at 74 weeks of age. Hens that preferred to use the range at early life were three times more likely to survive. Early range users were also 1.6 times more likely to become infected with gastrointestinal nematodes and showed significantly more frequent signs indicating spotty liver disease. Hens that preferred to stay in the shed during early life had a higher prevalence of Fatty Liver Syndrome and significantly less feather cover. In conclusion, hens that do not range during early life may benefit from additional management strategies to increase their likelihood of survival. Further investigations under controlled environmental conditions are warranted to quantify further the observed effects.

**Abstract:**

While free-range laying hens frequently experience health and welfare challenges, the contribution of range use towards these risks are largely unknown. The aim of this pilot study was to investigate the survival, health and welfare of commercial free-range laying hens and explore the association with early range use. Range use of 9375 Lohmann Brown hens housed within five flocks was assessed during 18–21 weeks of age and individual hens were classified as “rangers” (frequent range users), “roamers” (intermittent range users), and “stayers” (rare/no range users) were then subject to necropsy at 74 weeks of age. Rangers and roamers were three times and 2.4 times more likely to survive than stayers, respectively (*p* = 0.001). Overall, rangers had significantly better feather cover and more lesions associated with spotty liver diseases compared to roamers and stayers (*p* = 0.001). Similarly, rangers and roamers had a higher prevalence of *A. galli* infection and less frequent signs of fatty liver syndrome compared to stayers. Rangers had a higher proportion of hens with full ovary follicle production compared to stayers and roamers (*p* = 0.035). This information is highly relevant to consider the targeted support of different flock subpopulations to improve hen health and welfare, directly affecting farm profitability. Further research on other farms is warranted to investigate the transferability of the observed results.

## 1. Introduction

One of the main goals of free-range egg production is to optimise laying performance and egg quality while supporting hen health and welfare. However, opportunities provided in the free-range system allow for the expression of a variety of behaviours, interactions and physical experiences (e.g., being exposed to uncontrolled environmental conditions) which may impact hen health, welfare, and survival [1,2,3]. For example, free-range flocks are frequently diagnosed with Spotty Liver Diseases (up to 20% prevalence), high and low pathogenic avian influenza, fowl cholera, infectious laryngotracheitis, and salmonella (up to 53% prevalence), or gastrointestinal parasites (up to 100% prevalence) [4,5,6,7,8,9]. The compromised biosecurity due to the exposure of the hens to the outdoors and subsequently wild birds, rodents and other unfavourable environmental conditions (water puddles, defecated areas) are held primarily responsible for these problems [4,6,10]. However, it is currently remains unknown to what extent range usage as such may affect hen health or to what extent the outdoor exposure may support physical health as seen in other animal species [11]. For example, in grazing species, the impact of continuous movement on pasture is considered to be beneficial due to better air quality, reduced mental stress and/or a modulated immune system resulting in an increased resilience against infectious and systemic diseases [12,13,14]. In poultry, superior feather cover is one benefit frequently associated with range use [14,15]. This is despite the fact that feather pecking is of multifactorial origin and involves hen genetics, the environment (light intensity, stocking density, enrichment, flock uniformity), diet, and early life experience [16,17,18,19,20,21,22]. In contrast, hens with access to pasture are more often affected by grass impaction, while shed use, especially when equipped with an aviary system has raised welfare concerns regarding keel bone damage [1,23,24].

Many investigators described a non-uniform range usage where up to 21% of hens within a flock never accessed the outdoors within a given time period [25,26]. The development of these ranging subpopulations within one flock is not only affected by flock size, genetics, weather conditions and other external factors, but also by early life experience, individual hen preferences and time budgets [25,26,27]. We previously showed that hens of different early range use (18–21 weeks of age) peaked their egg production at different ages, where to 10% difference of laying performance was observed at 22 weeks of age as well as at 72 weeks of age [28]. However, farm management needs to consider not only laying performance and egg quality, but also hen house production which is affected by health and mortalities. Therefore, the aim of this study was to investigate the association between range use and hen survival as well as various health and welfare parameters.

## 2. Materials and Methods

### 2.1. Ethical Statement

All procedures carried out in this study were approved by the University of New England’s Animal Ethics Committee (AEC 16-087).

### 2.2. Animal Housing and Management 

A total of 5 flocks (Flocks A–E) each housing 40,000 Lohmann Brown hens were kept on the same commercial farm. In each flock, 3125 randomly chosen hens were leg-banded at the day of placement (at 16 weeks of age) for individual identification and then placed in partitioned cross-sections of the shed, allowing for the monitoring of individual range access using a Radio-Frequency Identification (RFID) system with technical details described and validated in Sibanda et al., (2020c) [29]. All hens experienced the same stocking density, diet, resources availability (number of next boxes, drinker lines, etc.), same management team and procedures, and all sheds had the same geographic orientation while being located next to each other. The shed was equipped with a tunnel ventilated system, curtain sides and pop holes along the entire length of the shed wall, which allowed constant air flow and temperature control. Manure was removed frequently using automated manure belts, preventing any ammonia build-up. However, ammonia levels were not measured. Details of the experimental set up are provided by Sibanda et al. (2020a) [27].

### 2.3. Subpopulation Classification

Based on the early range usage of the 3125 hens/flock during 18–21 weeks of age, individual hens were selected and arranged into 3 groups (stayers, roamers, and rangers) with 625 hens/group, leading to the study population of n = 1875 hens/flock for further investigation. “Stayers” referred to the flock population that spent the least days on the range (4.72% ± 0.31% of their available days), “roamers” included those hens that occasionally visited the range (40.6% ± 0.74% of their available days), and “rangers” spent the most days on the range (77.8% ± 0.56% of their available days). These percentages equated to 2.09 ± 0.15 min/hen/day (stayers), 18.3 ± 0.51 min/hen/day (roamers), and 55.6 ± 0.76 min/hen/day (rangers) on the range. While initially 9375 hens were tracked (1875 hens in each of the 5 flocks), 4085 hens were excluded from the health and welfare statistical analysis at 74 weeks of age due to lost or malfunctioning RFID tags as well as hen mortalities. Details obtained from the surviving hens where range use could be recorded until 74 weeks of age are shown in Table 1. Over the duration of the study, hen mortalities were recorded by farm staff, noting the individual identification number and the date of death. A total of 6729 hens were included in the mortality analysis.

### 2.4. Health and Welfare Assessment

At 74 weeks of age, flocks were subject to depopulation which allowed for individual evaluation of all surviving hens for gastro-intestinal helminths, keel bone damage, liver health, plumage condition and ovary egg follicle production. After the hens were captured, the identification number was recorded and the plumage damage for each body region (neck, chest, wing, back, and vent/cloaca) of individual hens was visually assessed using a 4-point system modified from Tauson et al. (2005) [30], where 1 indicated no feather coverage, 2 indicated feather loss with more than 50% of the skin covered with feathers, 3 indicated moderate feather loss, and 4 indicated full feather coverage. Hens were then sacrificed by cervical dislocation before being subject to gross necropsy. After opening the carcass of the hens, keel bones were inspected visually and palpatory and scores assigned according to the severity of keel bone damage (score 0 = no damage; score 1 = minor bone damage; 2 = severe damage). The keel bone damage scores included keel bone deviation (lateral or dorsal deviation) and fractures. The presence or absence of miliary spots on the liver and the severity of fatty liver disease was categorized using a score of 0 = normal physiological liver colour; 1 = evidence of mild fatty liver and 2 = evidence of severe fatty liver. A subsample of hens with miliary spots of the liver were subject to diagnostic PCR testing where liver tissue (approximately 2 g), bile fluid (aspirated with needle and syringe), a caecal swab, and a cloacal swab were evaluated. To investigate the prevalence of gastrointestinal parasites, the length of the small intestine was opened and the presence or absence of cestodes and *Ascaridia galli* (*A. galli*) noted. Evaluation of the follicle ovulation stage of the ovaries was performed by visual scoring where 1 indicated no active follicles, 2 indicated the presence of follicles in late regression, 3 indicated the presence of follicles in early regression, and 4 indicated full follicle production. All observations of all hens were performed by the same investigator.

### 2.5. Statistical Analysis

Data were analysed using JMP Statistics software (v14 IBM SAS Institute Inc., Cary, NC, USA, 1989–2007) and statistical significance set at *p* < 0.05 unless stated otherwise. Four sets of data analysis are presented: firstly, mortality data were investigated using Kaplan-Meyer survival analysis including the Log-rank (Mantel-Cox) test and the Gehan–Beslow–Wilcoxon test with censored data based on day of death. This was followed by the calculation of the odds ratios for survival. Furthermore, odds ratios were calculated for each health parameter obtained at 74 weeks of age using a nominal regression model. The keel bone and fatty liver scores were modified and reported as the presence and absence of findings regardless of the severity to calculate the odds ratio. Secondly, descriptives of flock and subpopulation regarding keel bone damage, cestodes and *A. galli* infestations, spots on the liver, Fatty Liver Syndrome, egg follicle, and feather (neck, chest, wing, back and vent) are presented where the individual hen (n = 5290) was used to describe the flock and group proportions. In all parameters, the percentage proportion of hens with different scores in each treatment group was calculated using the total number of hens in each treatment group while the total proportion was calculated using the total number of hens in the flock. Thirdly, to investigate the impact of early range use on health parameters at the end of lay, the average score of all flocks was analysed using a restricted maximum likelihood (REML) model with the sub-group as a fixed factor, flock as random factor and the individual hen as statistical unit. The REML was used to reduce the effect of the non-independency of data points and the unbalanced design. Random effects in the REML allow for the control of non-independence by constraining non-independent ‘experimental units’ to have the same intercept and slope [31,32]. Furthermore, REML is a substitute to GLIMM that estimates the random-effect parameters in relation to the fixed-effect parameters and the REML estimates of standard deviations are less biased compared to GLIMM. Fourthly, to understand whether there was a difference between the pooled flock mean score of all parameter scores, an additional analysis was carried out to compare the overall population mean of the subpopulations using analysis of means for proportions–transformed ranks (ANOM).

## 3. Results

### 3.1. Mortality

**Figure 1:**
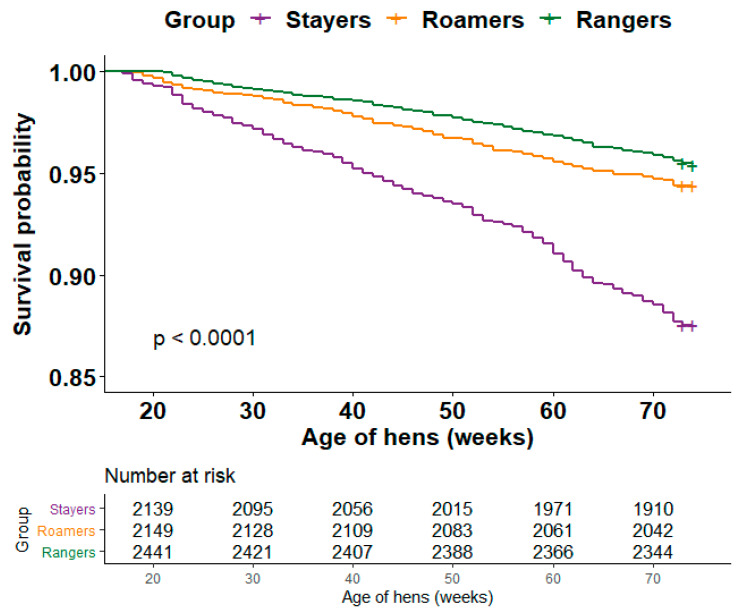
The survival analysis using the Kaplan-Meier estimates for 5 flocks of stayers, roamers and rangers. Censoring is indicated by the horizontal line tic mark.

Survival for rangers, roamers and stayers differed significantly (*p* < 0.001; Figure 1). Rangers were 2.99 times more likely to survive compared to the stayers (*p* = 0.001), while the roamers were 2.39 times more likely to survive compared to the stayers (*p* = 0.001; Table 2). There was no significant difference between the survival of rangers and roamers (*p* = 0.098, Table 2).

### 3.2. Gastrointestinal Helminths

**Figure 2:**
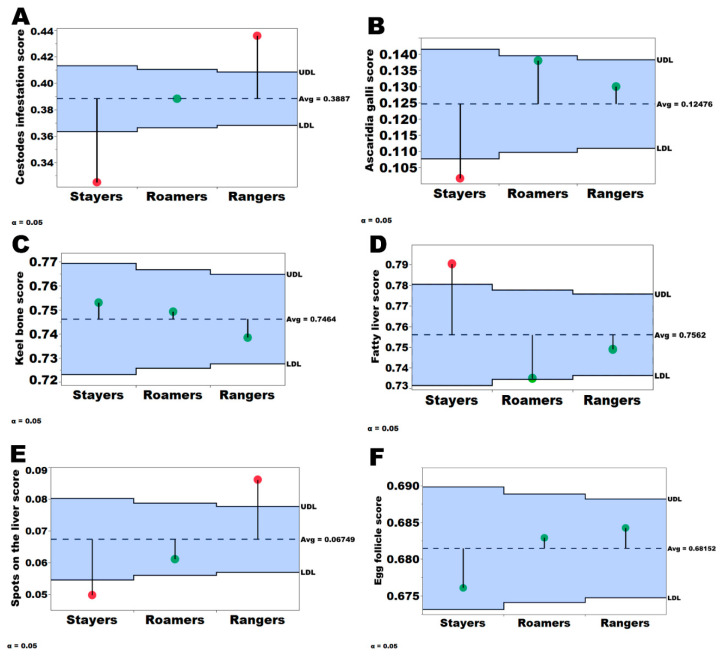
Analysis of means for proportions for *A. galli* (**A**), cestodes (**B**), keel bone (**C**), fatty liver (**D**), spots on the liver (**E**) and egg follicle condition score (**F**). The solid black lines indicate the upper decision line (UDL) and the lower decision line (LDL) at the 95% confidence level. The dashed black line represent the population mean score. The red dots at the end of the needle indicate a significant difference (*p* < 0.05) from the population mean, while the green dots at the end of the needle indicate no statistical significance.

The prevalence of gastrointestinal helminths is shown in Table 3 and Table 4. Rangers and roamers were 1.32 and 1.41 times more likely to be infected with *A. galli* (*p* = 0.016; 0.001), respectively compared to stayers (Table 2). Rangers were 1.60 and 1.22 times more likely to be infected by cestodes compared to stayers and roamers (*p* = 0.001), furthermore roamers were 1.31 more likely to be affected by cestodes compared to stayers. There was a significant flock*group interaction for cestodes infestation (*p* = 0.01; Table 4, Figure S1) while no interaction for *A. galli* could be observed (*p* = 0.244). Stayers had up to 3% less *A. galli* worms (*p* = 0.001) compared to rangers while rangers had a 10.8% and 19.3% higher prevalence of cestodes compared to roamers and stayers, respectively (*p* = 0.001). Furthermore, stayers had a significantly lower *A. galli* score compared to the roamers, rangers, and the pooled flock average (*p* < 0.05, Figure 2, Table 4). When comparing the pooled flock mean score to the group means, the stayers had a significantly lower cestodes infestation score compared to the flock mean while the rangers had a significantly higher cestodes infestation score compared to the flock mean (*p* < 0.05; Figure 2).

### 3.3. Keel Bone Damage

Stayers, roamers, and rangers had a similar likelihood to be affected by keel bone damage (*p* > 0.050; Table 2). There was a flock * group interaction effect on keel bone damage score (*p* = 0.018, Table 4) where hens of Flock A had a significantly higher keel bone score compared to all the remaining flocks, and hens of Flock B had the highest proportion of hens with no keel bone damage (63.1%; *p* = 0.001; Table 3). There were no significant differences in keel bone score between the groups of rangers, roamers, or stayers (*p* = 0.523; Table 4). Overall he prevalence of hens with any keel bone damage, regardless of the severity (minor or severe damage), ranged from 0% to 69.7% among subgroups from different flocks.

### 3.4. Liver Health

Diagnostic PCR confirmed the presence of Campylobacter hepaticus, the causative agent of Spotty Liver Disease in every sample taken. The results of the prevalence and score of liver conditions are presented in Figure 2 and Table 3 and Table 4. The rangers were 1.80 and 1.45 times more likely to be affected by spots on the liver compared to the stayers and roamers (*p* = 0.049; Table 2), while the roamers had the same likelihood as stayers (*p* = 0.934). Rangers of Flock A had a higher spot on the liver score compared to the roamers and rangers in the same flock (*p* = 0.001). When comparing hens affected by spots on the liver, a total of 4.99% of stayers (pooled for all flocks) had spots on their livers, while 6.12% and 8.62% of the roamers and rangers were affected (Table 3). Rangers had significantly higher scores compared to the flock mean (*p* < 0.05, Figure 2).

The stayers were 1.26 and 1.36 times more likely to be affected by the Fatty Liver Syndrome compared to roamers and rangers (*p* < 0.001; Table 2). There was a flock * group interaction regarding the score of Fatty Liver Syndrome (*p* < 0.01; Table 4) where stayers in Flock B, C, and D presented a higher fatty liver score compared to roamers and rangers (*p* = 0.001). When all the five flocks were pooled together, up to 10.6% and 6.01% of the hens had mild and severe Fatty Liver Syndrome, respectively. Overall, a total of 15.2% of the 5290 necropsied hens from the five flocks were affected by Fatty Liver Syndrome.

### 3.5. Plumage Condition

**Figure 3:**
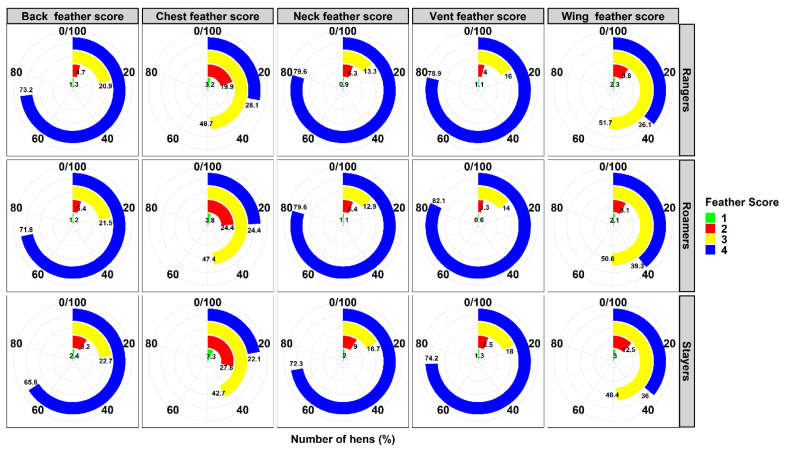
Descriptive prevalence of plumage condition observed in 5290 commercial laying hens that were classified as stayers, roamers and rangers. The figure summarises the various ranging groups pooled from 5 flocks. A score of 4 indicates full feather cover, 3 indicates moderate feather cover, 2 indicates feather loss with more than 50% of the skin covered with feather, and 1 indicates severe feather loss with less than 50% of the skin covered with feather [23].

The prevalence of plumage condition of different body parts is shown in Figure 3. Rangers presented a better neck, chest, back and vent plumage condition score compared to stayers (all *p* < 0.001; Table 3). Rangers were not statistically different when comparing neck and back feather score when equated to roamers but roamers had significantly better vent cover scores (3.78 ± 0.01) compared to stayers (3.66 ± 0.02; *p* = 0.001), and comparable good cover compared to rangers (3.73 ± 0.01; *p* > 0.05). The stayers and rangers had similar wing plumage condition scores of 3.17 ± 0.02 and 3.22 ± 0.02, which was statistically significantly lower compared to the roamers (3.27 ± 0.02; *p* = 0.001). There was a significant flock*group interaction (*p* = 0.001) when considering the cumulative feather score. Flock A and Flock B had the highest overall feather score while in Flocks C, D, and E, the stayers had significantly lower plumage condition compared to rangers.

### 3.6. Egg Follicle Production

A significant interaction was observed between flock and group regarding egg follicle scores, where in Flock B & D rangers had significantly more egg follicles in full production compared to stayers, but in Flock E rangers had significantly less egg follicles in full production (Table 4, Figure S2). When the flocks were pooled, 93.2% of the stayers were in full production compared to 94.2% and 94.4% of the roamer and ranger, respectively (Table 4). Overall, stayers had the highest percentage of hens with follicles in early regression (4.06%) compared to rangers (3.14%) and roamers (2.69%; Table 5). When investigating individual flocks, this significant difference could be observed in Flock B, C, and E. Although, more than 90% of hens in every flock were in full production, hens obtained from Flock D had more hens in full production (95.4%) compared to hens obtained from Flocks B (90.4%) and C (92.7%).

## 4. Discussion

Stayers were three times more likely to experience overall mortality (*p* = 0.001; Table 2). This result is surprising when taking the debates about biosecurity of free-range hens into account, where the exposure to the outdoors is frequently suspected to be responsible for higher flock mortalities [2,33]. Hens with reduced body condition might have preferred to seek shelter within the shed, and therefore the health status of the hens at the time of placement might have determined its assignment into the stayer group, rather than the hens dying as a consequence of staying indoors. While every individual hen was manually handled, inspected and leg-banded at the time of placement, no obvious signs of diseases could be observed at that time. However, poultry are known for their silent suffering where the observation of clinical signs is commonly unspecific (ruffled feathers), and they usually appear only a few days before death [34]. Unfortunately, it was beyond the scope of this study to investigate the mortality reasons of the hens that died before 74 weeks of age and therefore causation cannot be determined. Depending on the causation, further research is highly warranted to explore options for early intervention which minimise the losses.

Exposure and infestation of several infectious diseases could be observed in the investigated flocks: the prevalence of gastrointestinal nematodes ranged from 0–57.4% (Table 3). The frequent observation of nematodes and cestodes especially in free-range systems has resulted in classifying gastrointestinal parasites as re-emerging diseases [35,36,37]. In agreement with previous investigators, a relatively high variation of the prevalence could be observed amongst flocks, which may reflect the impact of environmental conditions/season [35]. Surprisingly, despite stayers, rangers, and roamers being subject to the same flock management conditions and having the same resource opportunities available, their chosen location at the beginning of lay significantly affected parasite infestation 50 weeks later. Rangers were 1.32 times more likely to be infected with *A. galli* compared to stayers, respectively [4]. Exposure to excreta containing embryonated parasite eggs provided by wild birds could have increased the prevalence of helminth infection of the rangers. However, exposure to embryonated *A. galli* eggs could have been expected to be equally present in range soil and in the indoor bedding material, as both resources would have been accessed by egg-shedding hens. With the stocking density being even higher indoors, and a higher hen traffic in the litter area where hens would travel through to access the range/aviary system can be observed, one may have even suspected that indoor hens would be more severely impacted by *A. galli*. On the other hand, hens that spent the majority of their time indoors would have used not only the litter area, but also the aviary system, where two manure belts, located at the top and bottom tier prevented hen exposure to excreta and subsequently interrupted the infection cycle of *A. galli* [38]. We previously showed that hens that chose their location on the top of the aviary system rarely access the lower tier, let alone the range [27]. These hens, which would represent most of the stayer population, would therefore experience similar conditions than caged hens in respect to the methods and frequency of manure collection. As a result, the prevalence of *A. galli* and infection scores in these stayer populations was less than the prevalence observed in the roamers and rangers (Table 4), indicating the possibility of effective interventions. Regardless the range usage between 21–74 weeks of age, the early life behaviour obviously impacted outcomes 50 weeks later, indicating a substantial contribution to overall hen health. In fact, it might have been parasite exposure and subsequent stimulation of the immune system, which may have increased the resilience of hens towards other infectious diseases [39]. For example, surviving rangers were 1.80 and 1.45 times more likely to be presented with miliary spots on the liver, indicating current or previous infection with Spotty Liver Diseases. The causative agent, *C. hepaticus* had previously been identified in a variety of vectors including access to insects, and soil [40,41]. With these vectors being more likely to be present on the range, it is understandable that the prevalence of Spotty Liver Diseases was significantly higher in rangers and roamers compared to stayers. It is surprising that, despite the fact that all hens were housed in the same shed environment, the groups were significantly differently affected. While one reason might be limited transmission amongst hens within the shed, one may also speculate that those affected stayers might have experienced fatal consequences. As discussed above, unfortunately the cause of the hens that died is unknown but further research investigating highly warranted to answer those relevant questions. Interestingly, in three of the five flocks, significantly more stayers exhibited liver discoloration compared to roamers and rangers, indicating a higher prevalence of Fatty Liver Syndrome. Clinical symptoms of Fatty Liver Syndrome are usually not seen in live birds and hens die peracute due to haemorrhage and liver capsule rupture [42,43]. Fatty Liver Syndrome has previously been shown to be most common in caged hens where 74% of the mortality was caused by the Fatty Liver Syndrome compared to 5% and 0% of hens from barn and free-range housing [44]. While stayers might have been less active in general, they would have also been in closer proximity to the feeder chains, which may have resulted in increased feed intake further promoting fat/energy deposition in the liver [45].

The significant flock by group interaction in egg follicle scores observed in this study indicates that the effect of range use was more apparent in some flocks compared to others. Rangers had higher follicle scores indicating more consistent egg production in Flock B, D, and E while no difference of follicle production could be observed between stayers, roamers and rangers in Flock A and C. The causation of flock difference warrants further investigation. We previously showed in the same flock that, when obtaining the deposited eggs and calculating the laying percentage in 10-weekly intervals, the overall laying percentage of stayers was significantly higher compared to roamers and rangers at 72 weeks of age [28]. This indicated that the method of ovary inspection to estimate egg production might not have been sensitive enough to detect differences relevant to the economic considerations of the egg producer. More importantly, the significantly higher mortality of stayers during their entire housing period in the present study puts the difference of superior laying percentage/group observed in the previous study in a new perspective. While stayers may have produced a significantly higher laying percentage at the end of lay, the economic benefit is likely to be irrelevant due to the lower absolute number of survivors. It remains unknown if the higher mortality of the stayers was linked to the higher incidence of Fatty Liver Syndrome. However, due to high prevalence of Fatty Liver Syndrome in stayers, these hens may benefit from prolonged physical exercise and nutritional supplementation, such as choline, methionine, vitamins, and betaine, to improve lipid metabolism to reduce the severity and impact of the fatty liver on the systemic body function [16,46,47,48].

The hens that used the outdoor range more frequently at early lay had a better feather score compared to the infrequent range users, which is similar to the result obtained by Bestman et al. (2003) [49] where 66% of the hens that accessed the range did not show signs of severe feather loss. Many researchers have noted the better feather cover of rangers compared to stayers [49,50] while Hartcher et al. (2016) [51] did not observe an association between range use and plumage condition. The proportion of hens with full plumage cover ranged from 88.2% to 94%, which might be due to the fact that more than 80% of the hens used the range. Furthermore, the hens from all groups experienced poor plumage cover on the chest and on the wings and this may be due to increased wear and tear during perching, feeding, or resting where the breast might have been placed more frequently on the shed equipment or the bedding material [51,52,53,54].

There was an overall high variation in keel bone damage which was similar to results obtained by others investigating non-caged housing systems [55,56,57,58]. The hens in this study were exposed to the aviary system not only during lay, but also when being reared and it had been frequently noted that this increased the risk of falling or of failed landings when exploring the available areas [59,60]. One may assume that stayers, spending more time in the aviary system, would have a higher exposure to potential damaging aviary structures and subsequently a higher likelihood and incidence of keel bone damage whereas free horizontal space on the range could have been a safer environment. However, there was no difference between the keel bone prevalence or severity of stayers, roamers, and rangers. Reasons for this may include that either keel bone damage could have been present at the time of hen placement (obtained in the rearing facilities), occurred within the first few weeks of placement before hens were allowed to access the range, or occurs regardless range utility given the fact that all hens use the aviary system for some time during the day [61]. Furthermore, keel bone damage may be unrelated to landing impact rather representing greenstick fractures as well as being associated with calcium metabolism, which might have affected all hens of the investigated flock equally [62]. The results of the present study indicate that range access has no additional benefit in preventing keel bone damage and that modification of the aviary system such as the use of soft perches or terrace-designed aviaries may currently provide the best approach for reducing/preventing keel bone damage [59].

As mentioned before, it is a major limitation of this study that the lack of data obtained at hen placement does not allow us to investigate causation. Investigation of health parameters of individual hens at placement and at several time points during their laying cycle before depopulation would allow for more applied recommendations for egg producers to improve and maintain hen health and welfare. Resource occupancy combined with time series data might be indicators of health, welfare, and productive performance of free-range laying hens. Furthermore, these time series could then be used to predict the effect of resource usage on the health and welfare of the free-range laying hens. However, it is remarkable that early range use was still associated with significantly different outcomes 50 weeks later, despite a variety of factors that could have intervened during this time and despite the fact that many stayers become rangers later in life [28]. To our best knowledge, this is the most comprehensive study that investigated the effect of early range use and hen health and welfare on a commercial scale to date and it subsequently provides strong justification for further investigation due to the significant differences in mortality with a large economic impact.

## 5. Conclusions

In this pilot study, hen health and welfare at 74 weeks of age differed significantly depending on early range use, which suggests that early range usage could potentially be used as a predictor of these outcomes, which might further be modified if targeted management decisions would apply. Despite the fact that stayers, ranges and roamers have the same resources available and were exposed to the same microclimate (dust, feed, and water lines, etc.), the prevalence of infectious diseases, such as Spotty Liver Diseases or intestinal parasites, differed significantly amongst these groups. This suggest that intervention can successfully reduce the transmission of these diseases within the flock. Despite the higher biosecurity risk associated with range use, rangers had a significantly higher chance of survival, according to several better health and welfare indicators. Further investigation of the causation could result in robust methods to predict overall health and welfare and allow for effective intervention methods.

## Figures and Tables

**Table 1:** Mean (±SEM) duration of range use, number of visits, number of days on the range during early life (18–21 weeks) and whole life (18–72 weeks) of the stayers, roamers and rangers.

Flock	Groups	Number of Hens with Full Dataset Available	Duration of Range Use (Min/Hen/Day)		Number of Range Visits (Visits/Hen/Day)		Mean Days on the Range/Hen		Percentage Days on the Range/Hen	
			18–21 Weeks	18–74 Weeks	18–21 Weeks	18–74 Weeks	18–21 Weeks	18–74 Weeks	18–21 Weeks	18–74 Weeks
**Flock A**	Stayers	298	4.19 ± 0.5	33.7 ± 0.9	0.57 ± 0.09	2.69 ± 0.06	0.725 ± 0.1	117.3 ± 3.4	4.26 ± 0.3	43.5 ± 1.3
	Roamers	404	39.9 ± 1.5	48.2 ± 1.0	1.82 ± 0.05	2.64 ± 0.03	6.883 ± 0.1	155.4 ± 3.1	40.5 ± 0.7	57.6 ± 1.1
	Rangers	409	83.6 ± 1.6	60.0 ± 0.9	2.38 ± 0.03	2.43 ± 0.02	13.20 ± 0.1	181.8 ± 2.9	77.7 ± 0.6	67.3 ± 1.1
**Flock B**	Stayers	362	0.77 ± 0.2	23.1 ± 0.9	1.19 ± 0.05	1.09 ± 0.05	2.197 ± 0.2	207.0 ± 2.9	8.45 ± 0.7	73.7 ± 1.0
	Roamers	380	4.91 ± 0.4	24.4 ± 0.7	1.43 ± 0.04	1.32 ± 0.04	3.501 ± 0.1	204.2 ± 3.1	13.5 ± 0.5	72.7 ± 1.1
	Rangers	401	36.5 ± 1.2	36.3 ± 0.8	1.82 ± 0.04	1.79 ± 0.04	11.95 ± 0.2	211.7 ± 2.8	46.0 ± 0.9	75.4 ± 1.0
**Flock C**	Stayers	296	1.15 ± 0.4	35.3 ± 1.5	0.27 ± 0.04	3.37 ± 0.10	2.680 ± 0.2	109.6 ± 3.4	11.2 ± 1.0	73.3 ± 1.0
	Roamers	375	7.29 ± 0.4	33.6 ± 1.1	1.72 ± 0.05	3.53 ± 0.08	5.520 ± 0.1	166.3 ± 3.9	23.0 ± 0.6	50.4 ± 1.2
	Rangers	444	51.6 ± 1.9	45.0 ± 1.9	3.26 ± 0.06	4.10 ± 0.07	16.40 ± 0.1	217.1 ± 3.1	96.8 ± 0.4	65.8 ± 0.9
**Flock D**	Stayers	253	1.58 ± 0.1	35.3 ± 1.3	1.89 ± 0.22	4.52 ± 0.15	3.626 ± 0.2	110.9 ± 3.3	12.9 ± 0.6	37.8 ± 1.1
	Roamers	395	20.7 ± 0.5	48.3 ± 1.2	4.03 ± 0.12	4.70 ± 0.09	13.26 ± 0.2	193.8 ± 3.2	47.3 ± 0.8	66.1 ± 1.1
	Rangers	386	66.5 ± 1.2	55.4 ± 1.2	5.38 ± 0.10	5.49 ± 0.10	23.32 ± 0.2	226.2 ± 2.8	83.3 ± 0.6	77.2 ± 1.0
**Flock E**	Stayers	294	1.92 ± 0.3	24.3 ± 1.1	1.00 ± 0.09	2.95 ± 0.09	3.132 ± 0.2	81.00 ± 2.6	4.9 ± 0.7	34.2 ± 1.1
	Roamers	227	15.5 ± 1.4	25.7 ± 1.5	2.57 ± 0.12	4.00 ± 0.12	8.554 ± 0.2	89.88 ± 3.1	40.7 ± 1.1	37.9 ± 1.3
	Rangers	366	44.4 ± 1.4	37.6 ± 1.0	4.25 ± 0.10	4.63 ± 0.08	18.24 ± 0.3	119.7 ± 2.2	86.9 ± 1.6	50.5 ± 0.9
**Pooled**	Stayers	1503	2.09 ± 0.2	30.3 ± 1.1	0.98 ± 0.10	2.92 ± 0.08	2.47 ± 0.2	124.8 ± 3.1	4.72 ± 0.3	52.5 ± 1.1
	Roamers	1781	18.3 ± 0.5	36.0 ± 1.1	2.31 ± 0.08	3.24 ± 0.09	7.53 ± 0.1	161.6 ± 3.3	40.6 ± 0.7	56.9 ± 1.2
	Rangers	2006	55.6 ± 0.8	46.9 ± 1.2	3.42 ± 0.07	3.69 ± 0.06	16.6 ± 0.2	190.8 ± 2.8	77.8 ± 0.6	67.2 ± 1.0

**Table 2:** Odds ratios for mortality, keel bone damage, cestodes and *A. galli* infection, fatty liver syndrome and spots on the liver comparing the results obtained from the various range groups (stayers, roamers, and rangers). The numbers in brackets represents the confidence interval at 95% of the odds ratios.

Comparing Groups		Mortality	Cestodes	*A*. *galli*	Fatty Liver Syndrome	Spots on the Liver	Keel Bone Damage
**Rangers vs.**	**Stayers**	0.33 (0.27, 0.42) ***	1.60 (1.40, 1.84) ***	1.32 (1.07, 1.63) **	0.79 (0.67, 0.95) ***	1.80 (1.36, 2.38) ***	1.01 (0.89, 1.16)
**Rangers vs.**	**Roamers**	0.80 (0.61, 1.04)	1.22 (1.07, 1.39) ***	0.93 (0.77, 1.13)	1.10 (0.90, 1.28)	1.45 (1.13, 1.86) ***	1.03 (0.90, 1.17)
**Roamers vs.**	**Stayers**	0.42 (0.33, 0.52) ***	1.31 (1.14, 1.52) **	1.41 (1.14, 1.75) **	0.73 (0.61, 0.89) ***	1.24 (0.92, 1.68)	0.99 (0.86, 1.13)
**Roamers vs.**	**Rangers**	1.25 (0.96, 1.63)	0.82 (0.72, 0.94) **	1.07 (0.89, 1.29)	0.93 (0.78, 1.11)	0.69 (0.54, 0.89) ***	0.97 (0.86, 1.11)
**Stayers vs.**	**Roamers**	2.39 (1.91, 2.99) ***	0.76 (0.66, 0.88) ***	0.70 (0.26, 0.87) **	1.36 (1.30, 1.63) ***	0.81 (0.60, 1.09)	1.01 (0.88, 1.16)
**Stayers vs.**	**Rangers**	2.99 (2.37, 3.78) ***	0.62 (0.54, 0.72) ***	0.76 (0.61, 0.94) **	1.26 (1.06, 1.50) ***	0.56 (0.42, 0.74) ***	0.98 (0.86, 1.12)

*p* > 0.05, ‘*’ *p* = 0.05, ‘**’ *p* = 0.001, ‘***’ *p* = 0.0001.

**Table 3:** Descriptives of the total number of hens (N) and the proportion of hens (%) with keel bone damage, cestodes, *A. galli* infection, spots on the liver and Fatty Liver Syndrome observed in stayers, roamers and rangers.

Flock	Group	Cestodes; N (%)	*A. galli*; N (%)	Minor Keel Bone Damage; N (%)	Severe Keel Bone Damage; N (%)	Spots on the Liver; N (%)	Mild Fatty Liver Syndrome; N (%)	Severe Fatty Liver Syndrome; N (%)
Flock A	Stayers	33 (11.1)	124 (41.6)	0 (0)	204 (68.5)	19 (6.38)	26 (8.72)	0 (0)
	Roamers	74 (18.3)	190 (47.0)	1 (0.25)	279 (69.1)	52 (12.9)	18 (4.46)	3 (0.74)
	Rangers	74 (18.1)	202 (49.4)	3 (0.73)	285 (69.7)	96 (23.5)	19 (4.65)	2 (0.49)
	Total	181 (16.3)	516 (46.4)	4 (0.36)	768 (69.1)	167 (15.0)	63 (5.67)	5 (0.45)
Flock B	Stayers	109 (30.1)	21 (5.80)	1 (0.28)	129 (35.6)	34 (9.39)	62 (17.1)	45 (12.4)
	Roamers	134 (35.3)	31 (8.16)	0 (0)	134 (35.3)	43 (11.3)	53 (14.0)	32 (8.42)
	Rangers	174 (43.4)	31 (7.73)	1 (0.25)	157 (39.2)	46 (11.5)	61 (15.2)	31 (7.73)
	Total	417 (36.5)	83 (7.26)	2 (0.18)	420 (36.7)	123 (10.8)	176 (15.4)	108 (9.44)
Flock C	Stayers	167 (56.4)	1 (0.34)	1 (0.34)	142 (48.0)	19 (6.42)	47 (15.9)	27 (9.12)
	Roamers	196 (52.3)	4 (1.07)	0 (0)	155 (41.3)	9 (2.40)	49 (13.1)	18 (4.80)
	Rangers	255 (57.4)	0 (0)	2 (0.45)	161 (36.3)	23 (5.18)	58 (13.1)	35 (7.88)
	Total	618 (55.4)	5 (0.45)	3 (0.27)	458 (41.1)	51 (4.57)	154 (13.8)	80 (7.17)
Flock D	Stayers	105 (41.5)	2 (0.79)	83 (32.8)	65 (25.7)	1 (0.40)	18 (7.11)	14 (5.53)
	Roamers	215 (54.4)	5 (1.27)	143 (36.2)	77 (19.5)	5 (1.27)	26 (6.58)	11 (2.78)
	Rangers	219 (56.7)	6 (1.55)	149 (38.6)	69 (17.9)	4 (1.04)	18 (4.66)	11 (2.85)
	Total	539 (52.1)	13 (1.26)	375 (36.3)	211 (20.4)	10 (0.97)	62 (6.00)	36 (3.48)
Flock E	Stayers	75 (25.5)	5 (1.70)	50 (17.0)	97 (33.0)	2 (0.68)	32 (10.9)	20 (6.80)
	Roamers	73 (32.2)	16 (7.05)	38 (16.7)	93 (41.0)	0 (0)	29 (12.8)	29 (12.8)
	Rangers	153 (41.8)	22 (6.01)	67 (18.3)	129 (35.3)	4 (1.09)	46 (12.6)	40 (10.9)
	Total	301 (33.9)	43 (4.85)	155 (17.5)	319 (36.0)	6 (0.68)	107 (12.1)	89 (10.0)
Pooled	Stayers	489 (32.5)	153 (10.2)	135 (8.98)	637 (42.4)	75 (4.99)	185 (12.3)	106 (7.05)
	Roamers	692 (38.9)	246 (13.8)	182 (10.2)	738 (41.4)	109 (6.12)	175 (9.83)	93 (5.22)
	Rangers	875 (43.6)	261 (13.0)	222 (11.1)	801 (39.9)	173 (8.62)	202 (5.8)	119 (5.93)
	Total	2056 (38.9)	660 (12.5)	539 (10.2)	2179 (41.1)	357 (6.75)	562 (10.6)	318 (6.01)

The numbers in brackets represents the proportion of hens in percentage.

**Table 4:** The mean ± SEM scores and statistical analysis of health and welfare parameters obtained from stayers, roamers and rangers.

		Cestodes Score ^†^	*A. galli* Score ^†^	Keel Bone Score ^¤^	Fatty Liver Score ^¢^	Spots on Liver ^†^	Accumulative Feather Score *	Egg Follicle Score ^§^
**Flock A**	Stayers	0.11 ± 0.03 ^h^	0.42 ± 0.02 ^b^	1.36 ± 0.05 ^a^	0.09 ± 0.03 ^f,g^	0.062 ± 0.014 ^c,d,e^	18.2 ± 0.15 ^a^	3.91 ± 0.03 ^a,b^
	Roamers	0.18 ± 0.02 ^g,h^	0.47 ± 0.01 ^a,b^	1.38 ± 0.05 ^a^	0.06 ± 0.03 ^g^	0.128 ± 0.012 ^b^	18.3 ± 0.13 ^a^	3.90 ± 0.02 ^a,b^
	Rangers	0.18 ± 0.02 ^g,h^	0.49 ± 0.01 ^a^	1.40 ± 0.05 ^a^	0.06 ± 0.03 ^g^	0.234 ± 0.012 ^a^	18.2 ± 0.13 ^a^	3.92 ± 0.02 ^a,b^
**Flock B**	Stayers	0.30 ± 0.02 ^e,f^	0.06 ± 0.01 ^c,d,e^	0.72 ± 0.05 ^c^	0.42 ± 0.03 ^a^	0.093 ± 0.013 ^b,c,d^	17.3 ± 0.14 ^b^	3.91 ± 0.02 ^a,b^
	Roamers	0.35 ± 0.02 ^d,e,f^	0.08 ± 0.01 ^c^	0.71 ± 0.05 ^c^	0.31 ± 0.03 ^a,b,c,d^	0.113 ± 0.012 ^b,c^	17.3 ± 0.13 ^b^	3.86 ± 0.02 ^a,b^
	Rangers	0.43 ± 0.02 ^b,c,d^	0.08 ± 0.01 ^c,d^	0.79 ± 0.05 ^b,c^	0.30 ± 0.03 ^a,b,c,d^	0.114 ± 0.012 ^b,c^	17.4 ± 0.13 ^b^	3.95 ± 0.02 ^a^
**Flock C**	Stayers	0.56 ± 0.03 ^a^	0.01 ± 0.02 ^e^	0.96 ± 0.05 ^b^	0.34 ± 0.03 ^a,b,c^	0.064 ± 0.014 ^c,d,e^	16.0 ± 0.15 ^d,e^	3.85 ± 0.03 ^a,b^
	Roamers	0.52 ± 0.02 ^a,b,c^	0.01 ± 0.01 ^d,e^	0.83 ± 0.05 ^b,c^	0.23 ± 0.03 ^c,d,e,f^	0.024 ± 0.013 ^e^	17.2 ± 0.13 ^b,c^	3.92 ± 0.02 ^a,b^
	Rangers	0.57 ± 0.02 ^a^	0.01 ± 0.01 ^e^	0.73 ± 0.04 ^c^	0.29 ± 0.03 ^b,c,d^	0.052 ± 0.011 ^d,e^	17.2 ± 0.12 ^b^	3.89 ± 0.02 ^a,b^
**Flock D**	Stayers	0.41 ± 0.03 ^c,d,e^	0.01 ± 0.02 ^c,d,e^	0.84 ± 0.06 ^b,c^	0.18 ± 0.03 ^d,e,f,g^	0.005 ± 0.015 ^e^	16.0 ± 0.16 ^d,e^	3.89 ± 0.03 ^a,b^
	Roamers	0.54 ± 0.02 ^a,b^	0.01 ± 0.01 ^d,e^	0.75 ± 0.05 ^b,c^	0.12 ± 0.03 ^e,f,g^	0.013 ± 0.012 ^e^	16.6 ± 0.13 ^c,d^	3.91 ± 0.02 ^a,b^
	Rangers	0.57 ± 0.02 ^a^	0.02 ± 0.01 ^c,d,e^	0.75 ± 0.05 ^b,c^	0.11 ± 0.03 ^e,f,g^	0.011 ± 0.012 ^e^	16.8 ± 0.13 ^b,c^	3.94 ± 0.02 ^a^
**Flock E**	Stayers	0.26 ± 0.03 ^f,g^	0.02 ± 0.02 ^c,d,e^	0.83 ± 0.05 ^c^	0.24 ± 0.03 ^b,c,d,e^	0.008 ± 0.014 ^e^	15.9 ± 0.15 ^e^	3.86 ± 0.03 ^a,b^
	Roamers	0.32 ± 0.03 ^d,e,f^	0.07 ± 0.02 ^c,d,e^	0.99 ± 0.06 ^b^	0.38 ± 0.04 ^a,b^	0.001 ± 0.016^e^	17.0 ± 0.17 ^b,c^	3.91 ± 0.03 ^a,b^
	Rangers	0.42 ± 0.02 ^c,d^	0.06 ± 0.01 ^c,d,e^	0.89 ± 0.05 ^b,c^	0.34 ± 0.03 ^a,b,c^	0.012 ± 0.013 ^e^	17.1 ± 0.14 ^b,c^	3.82 ± 0.02 ^b^
**Group**		0.001	0.004	0.532	0.001	0.001	0.001	0.518
**Flock**		0.001	0.013	0.123	0.001	0.001	0.001	0.001
Flock * Group		0.011	0.244	0.018	0.094	0.001	0.001	0.035
**R^2^ value**		0.095	0.289	0.069	0.048	0.067	0.07	0.007
**F ratio**		5.45	1.39	7.89	4.21	2.3	54	2.96

^a,b,c,d,e,f^ and ^g^ were used to identify statistical significance between groups (stayer, roamers, rangers) groups. ^†^ 0 indicates absent, 1 present. **^¤^** 0 indicates no keel bone damage, 1 minor damage 2 severe keel bone damage. **^¢^** 0 indicates physiologic normal liver, 1 mild Fatty Liver Syndrome, 2 severe Fatty Liver Syndrome. * 1 indicates no feather cover, 2 indicates feather loss with more than 50 % of the skin covered with feather, 3 indicates moderate feather loss and 4 full feather cover for each of the following regions: neck, breast, wing, back, tail. ^§^ 1 indicates no follicle, 2 late regression, 3 early regression, 4 full egg production.

**Table 5:** Descriptive of hens with different egg follicle characteristics of stayers, roamers and rangers in free range laying hens at 74 weeks of age in the five flocks. The numbers in brackets present the percentage proportion of hens.

Flock	Group	Egg Follicle Observation; N (%)			
		No Follicles	Late Regression	Early Regression	Full Egg Production
Flock A	Stayers	3 (1.01)	3 (1.01)	9 (3.02)	283 (95.0)
	Roamers	3 (0.74)	7 (1.73)	15 (3.71)	379 (93.8)
	Rangers	1 (0.24)	10 (2.44)	10 (2.44)	388 (94.9)
	Total	7 (0.63)	20 (1.80)	34 (3.06)	1050 (94.5)
Flock B	Stayers	0 (0)	3 (0.83)	24 (6.63)	335 (92.5)
	Roamers	9 (2.37)	6 (1.58)	11 (2.89)	354 (93.2)
	Rangers	2 (0.50)	1 (0.25)	12 (2.99)	386 (96.3)
	Total	11 (0.96)	10 (0.87)	47 (4.11)	1075 (90.4)
Flock C	Stayers	7 (2.36)	5 (1.69)	15 (5.07)	269 (90.9)
	Roamers	3 (0.80)	6 (1.60)	10 (2.67)	356 (94.9)
	Rangers	2 (0.45)	13 (2.93)	20 (4.51)	408 (92.1)
	Total	12 (1.08)	24 (2.15)	45 (4.04)	1033 (92.7)
Flock D	Stayers	5 (1.98)	2 (0.79)	6 (2.37)	240 (94.9)
	Roamers	6 (1.52)	1 (0.25)	15 (3.80)	373 (94.4)
	Rangers	2 (0.52)	4 (1.04)	7 (1.81)	373 (96.7)
	Total	13 (1.26)	7 (0.68)	28 (2.71)	986 (95.4)
Flock E	Stayers	11 (3.74)	3 (1.02)	7 (2.38)	273 (92.9)
	Roamers	6 (2.64)	1 (0.44)	5 (2.20)	215 (94.7)
	Rangers	16 (4.37)	8 (2.19)	5 (1.37)	337 (92.1)
	Total	33 (3.72)	12 (1.35)	17 (1.92)	825 (93.0)
Pooled	Stayers	26 (1.73)	16 (1.06)	61 (4.06)	1400 (93.2)
	Roamers	27 (1.52)	21 (1.18)	56 (3.14)	1677 (94.2)
	Rangers	23 (1.15)	36 (1.80)	54 (2.69)	1892 (94.4)
	Total	76 (1.44)	73 (1.38)	171 (3.23)	4969 (93.9)

## Supplementary Materials

The following are available online at https://www.mdpi.com/2076-2615/10/10/1911/s1, Figure S1: Flock * group interaction plots for the cestodes score, Ascaridia galli score, keel none score and total feather score, Figure S2: Flock * group interaction plots for fatty liver score, spots on the liver score and egg follicle score (C).
