# Supplementary material for: Managing Free-Range Laying Hens—Part B: Early Range Users Have More Pathology Findings at the End of Lay but Have a Significantly Higher Chance of Survival—An Indicative Study
Source: Animals (Basel). 2020 Oct 18;10(10):1911. doi: 10.3390/ani10101911 (PMC7603192; doi:10.3390/ani10101911)
Supplement: Supplementary file 1 [file animals-10-01911-s001.pdf]

Supplementary Materials

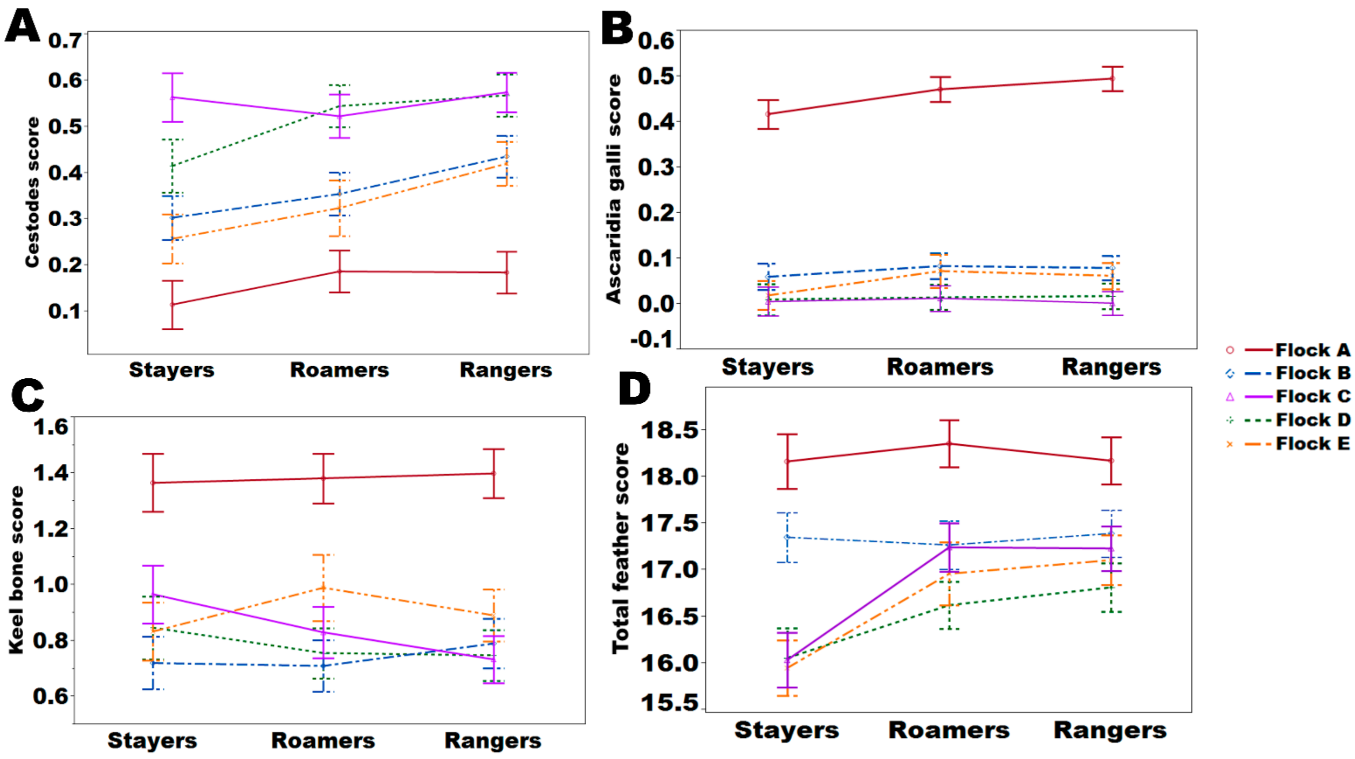

Figure S1. Flock * group interaction plots for the cestodes score (A), Ascaridia galli score (B), keel bone score (C) and total feather score (D). Cestodes and *A. galli* are represented by 0 indicating absence, 1 presence of the parasites. Keel bone scores are represented by 0 indicating no keel bone damage, 1 minor damage, 2 severe keel bone damage. Feather scores are represented by a score of 4 indicating full feather cover, 3 indicated moderate feather pecking, 2 indicates feather loss with more than 50 % of the skin covered with feather, and 1 indicates severe feather loss with less than 50 % of the skin covered with feather. A total of score from the neck, vent, wing, chest and back are presented.

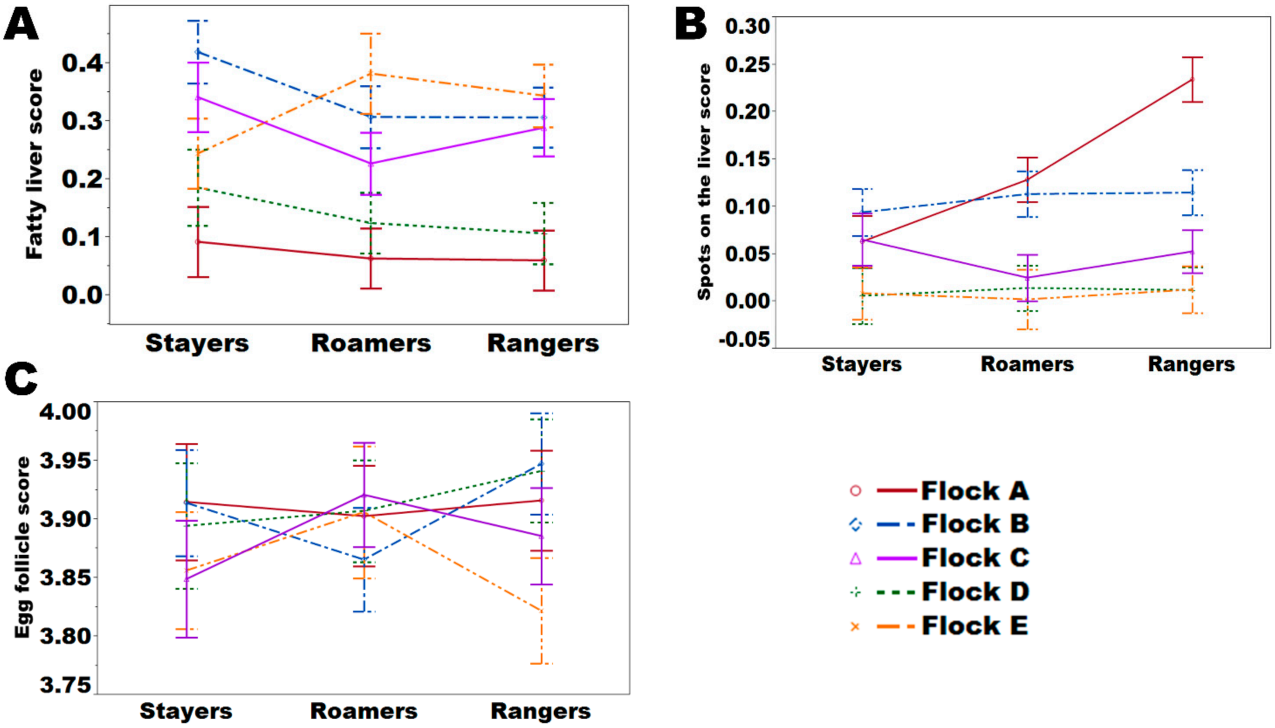

Figure S2. Flock * group interaction plots for fatty liver score (A), spots on the liver score (B) and egg follicle score (C). Spots on the liver is represented by a score of 0 indicating absent, 1 present. Egg follicle score is represented by a score of 1 indicating no follicle, 2 late regression, 3 early regression, 4 full egg production. Fatty liver score is represented by a score of 0 indicating physiologic normal liver, 1 mild Fatty Liver Syndrome, 2 severe Fatty Liver Syndrome. .
